# Live birth after Laser Assisted Viability Assessment (LAVA) to detect pentoxifylline resistant ejaculated immotile spermatozoa during ICSI in a couple with male Kartagener’s syndrome

**DOI:** 10.1186/s12958-018-0321-6

**Published:** 2018-02-05

**Authors:** Sinan Ozkavukcu, Ciler Celik-Ozenci, Esma Konuk, Cem Atabekoglu

**Affiliations:** 1 Department of Obstetrics and Gynecology, Ankara University School of Medicine, Center for Assisted Reproduction, Ankara Universitesi Tip Fakultesi Cebeci Hastanesi, Kadin Hastaliklari ve Dogum AD, ÜYTE Merkezi, Dikimevi-Ankara, Turkey; 2 Department of Histology and Embryology, Akdeniz University School of Medicine, Akdeniz Universitesi Tip Fakultesi Histoloji ve Embriyoloji AD, Konyaaltı-Antalya, Turkey

**Keywords:** Kartagener’s syndrome, Immotile cilia, Laser assisted viability assay, ICSI, Asthenozoospermia

## Abstract

Primary ciliary dyskinesia (PCD) is a rare, autosomal recessive disease with abnormalities in the structure of cilia, causing impairment of muco-ciliary clearance with respiratory tract infections, heterotaxia and abnormal sperm motility with male infertility. Here, with a comprehensive literature review, we report a couple with an infertility history of 9 years and three unsuccessful IVF treatments, where male partner has Kartagener’s Syndrome, a subtype of PCD, displaying recurrent respiratory infections, dextrocardia and total asthenozoospermia. His diagnosis was verified with transmission electron microscopy and genetic mutation screening, revealing total absence of dynein arms in sperm tails and homozygous mutation in the ZMYND10, heterozygous mutations in the ARMC4 and DNAH5 genes. Laser assisted viability assay (LAVA) was performed by shooting the sperm tails during sperm retrieval for microinjection, following detection of pentoxifylline resistant immotile sperm. Live births of healthy triplets, one boy and two monozygotic girls, was achieved after double blastocyst transfer.

**Electronic supplementary material:**

The online version of this article (10.1186/s12958-018-0321-6) contains supplementary material, which is available to authorized users.

## Introduction

Primary ciliary dyskinesia (PCD), is an uncommon (with a prevalence of 1/10,000), autosomal recessive genetic disorder that impairs the action of cilia in the lining of the respiratory tract and fallopian tube, as well as in the flagella of spermatozoa. Patients usually suffer from recurrent respiratory infections like chronic sinusitis, and bronchiectasis where situs inversus may accompany in 50% of the cases. Even though, PCD covers all congenital ciliary dysfunctions, the term Kartagener’s syndrome (KS) is used for describing the syndrome accompanied by situs inversus [1].

One of the major consequence of KS in males is infertility because of the vanished motility of spermatozoa. Mutations on genes that control the synthesis of inner and outer dynein arms or radial spokes cause the onset of KS and are used to diagnose the disease.

Kartagener’s syndrome results in immotile sperm production, males are incapable of achieving pregnancy through natural conception and the usage of intracytoplasmic sperm injection (ICSI) is indicated [2–4]. It is reported that motility of sperm in KS can be detected in some cases [5–9] and it can be enhanced using pentoxifylline [10, 11], or embryos can be grown from randomly selected immotile sperms with the help of assisted oocyte activation [12, 13]. Cases with the retrieval of testicular sperm were also reported [14–16]. The hypo osmotic swelling test (HOST) is the classical method to detect immotile but live spermatozoa [17], although test can be considered as detrimental, as the sperm cells are completely exposed to imbalanced osmotic conditions thus causing hypo-osmotic stress. Another technique, detecting the sperm tail flexibility by mechanical touching using ICSI pipette was also reported [18, 19]. Following experimental [20] and clinical [21, 22] reports of laser assisted sperm immobilization procedure, Aktan et al. have reported that, applying a single shot of laser on the tail of immotile spermatozoon causes an immediate tail curling, if the sperm is viable, possibly initiating a uniform damage on cell membrane that activate an influx towards the osmotic gradient [23].

Here we present a couple with male KS, demonstrating pentoxifylline-resistant total immotile spermatozoa which were selected by laser assisted viability assessment (LAVA) during ICSI and consequently achieved a normal pregnancy and live birth. In order to support the diagnosis, we performed a transmission electron microscopy (TEM) evaluation to visualize the axoneme ultrastructure in sperm tails and also a genetic assessment upon Kartagener’s panel for the possible mutations.

## Material and methods

Local ethical committee approval and informed consent from all individual participants included in the report was obtained.

Couple with an infertility history of 9 years referred to Ankara University Center for Assisted Reproduction with a desire to achieve a pregnancy. In another center, they have previously received three cycles of in vitro fertilization (IVF) treatments, one using testicular sperm, where only one resulted in biochemical pregnancy. TEM preparation was performed from ejaculate and sperm tails were evaluated, together with a donor semen as an internal control, in order to detect the presence of dynein arms according to a method previously reported [24]. One-step eosin-nigrosin viability test [25] was conducted to detect sperm viability. In order to support the diagnosis, genetic mutation screening was performed on patient’s whole blood sample, according to Kartagener’s panel using next generation sequencing (NGS) (Prevention Genetics, Marshfield, WI, United States).

In April 2016, controlled ovarian stimulation was planned using the GnRH antagonist method. A gonadotropin-releasing hormone analogue triggering together with the injection of 1500 U of hCG was administered when the dominant follicle reached a diameter of 20 mm. Vaginal ultrasound-guided oocyte retrieval was conducted under general anesthesia, 36 h after the hCG injection. To prepare spermatozoa for ICSI, liquefied semen was centrifuged at 400 *g* for 10 min and washed twice with sperm washing medium (SpermRinse, Vitrolife). ICSI dishes were prepared using a 3-(N-morpholino) propanesulfonic acid (MOPS) -buffered medium (G-MOPS, Vitrolife) under mineral oil (OVOIL, Vitrolife) by making two different large sperm pools. Washed sperm suspensions were deployed equally into the sperm pools on the ICSI dish. Both pools were investigated and scanned for spermatozoa under an inverted microscope at 400× magnification. Spermatozoa were evaluated as total immotile on all light microscopic fields observed; therefore, pentoxifylline was added into one of the sperm pools at a final concentration of 1 mg/mL [11]. After 15 min of incubation, pentoxifylline-applied-pool was screened for motile spermatozoa, nevertheless no motile spermatozoa were detected (Additional file 1: Video S1). Depending on the high viability score in eosin-nigrosin staining, LAVA was planned on the spermatozoa deployed in the second pool. Immotile sperm population was scanned and the ones with a visible tail structure were detected under 400× magnification. Distal piece of sperm tails were aligned with the target on the laser software (Cronus 3, Research Instruments) with the smallest possible hole diameter created by the pulse of 350 μs, and hit using a noncontact diode laser with an output wavelength of 1480 nm (Saturn 3, Research Instruments). The laser unit was coupled to an inverted microscope (Eclipse TE2000-U; Nikon Corporation, Tokyo, Japan) having a heated stage and micromanipulation devices (TransferMan NK2, Eppendorf, Hamburg, Germany) for sperm manipulation and ICSI. Spermatozoa that responded to the laser shot by a curling reaction of the tail or a sudden displacement of head were considered to be likely viable and retrieved for oocyte microinjection as shown in the visual demonstration (Additional file 2: Video S2). After ICSI, injected oocytes were washed and transferred into pre-incubated (overnight to maintain pH value of 7.27 and 5% O_2_ concentration) embryo culture medium (G-TL, Vitrolife) until pronucleus (PN) check, for confirmation of fertilization. Post-ICSI zygotes were evaluated after 17 h. Fertilized oocytes were transferred into a fresh pre-incubated culture medium prepared on a time-lapse 16-μ well group culture dish (Primo Vision Culture Dish, Vitrolife) and dish was loaded on a time-lapse microscope (Primo Vision™ Time-Lapse System, Vitrolife), installed in a multi-gas incubator (MCO-5 M-PE, Panasonic Healthcare, Tokyo, Japan). Time-lapse incubation was set for 5 days (up to embryo day 6) and time lapse images to be taken in every 7 min as shown in the video (Additional file 3: Video S3).Additional file 1: Video S1.Application of pentoxifylline. Lack of motility before and after pentoxifylline application on spermatozoa. (MP4 13369 kb)Additional file 2: Video S2.Application of LAVA. Immotile spermatozoa with a visible tail were selected visually (yellow arrow) and a single laser shot was performed on the distal region of the tail (red dot). Spermatozoa that have responded to the laser shot by a curling reaction of the tail or sudden head displacement were considered to be likely viable and retrieved for oocyte microinjection. (MP4 29842 kb)Additional file 3: Video S3.Developmental pattern of embryos. Time-lapse video of fertilized embryos of the couple with male Kartagener’s syndrome in extended culture. Two blastocysts were transferred on day 5. (Video was rendered in double speed). (MP4 23559 kb)

## Results

**Fig. 1:**
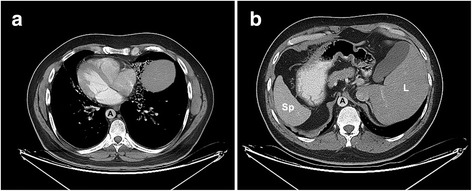
Thoracic and abdominal computerized tomography images. Thoracic (**a**) and abdominal (**b**) computerized tomography images displays dextrocardia (**a**) and situs inversus viceralis (**b**) in reported patient A: aorta, Sp: spleen, L: liver

**Fig. 2:**
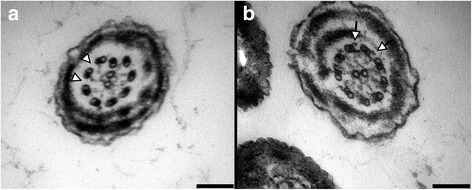
Transmission electron microscopy images. Transmission electron microscopy images reveal complete absence of dynein arms within the sperm tails in patient’s sample (**a**, arrowheads), while control sperm tails present normal dynein formation in donor’s sample for internal control (**b**, arrows). Scale bars indicate 0.1 μm

**Fig. 3:**
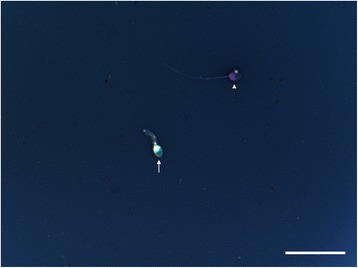
Eosin-nigrosin staining for viability assessment. Eosin is used to mark dead cells which uptake the stain through damaged, porous membranes and appear red or pinkish (arrowhead), where live spermatozoa that hinder eosin to penetrate head region because of their intact membrane, appear white (arrow). Scale bar indicates 50 μm

**Fig. 4:**
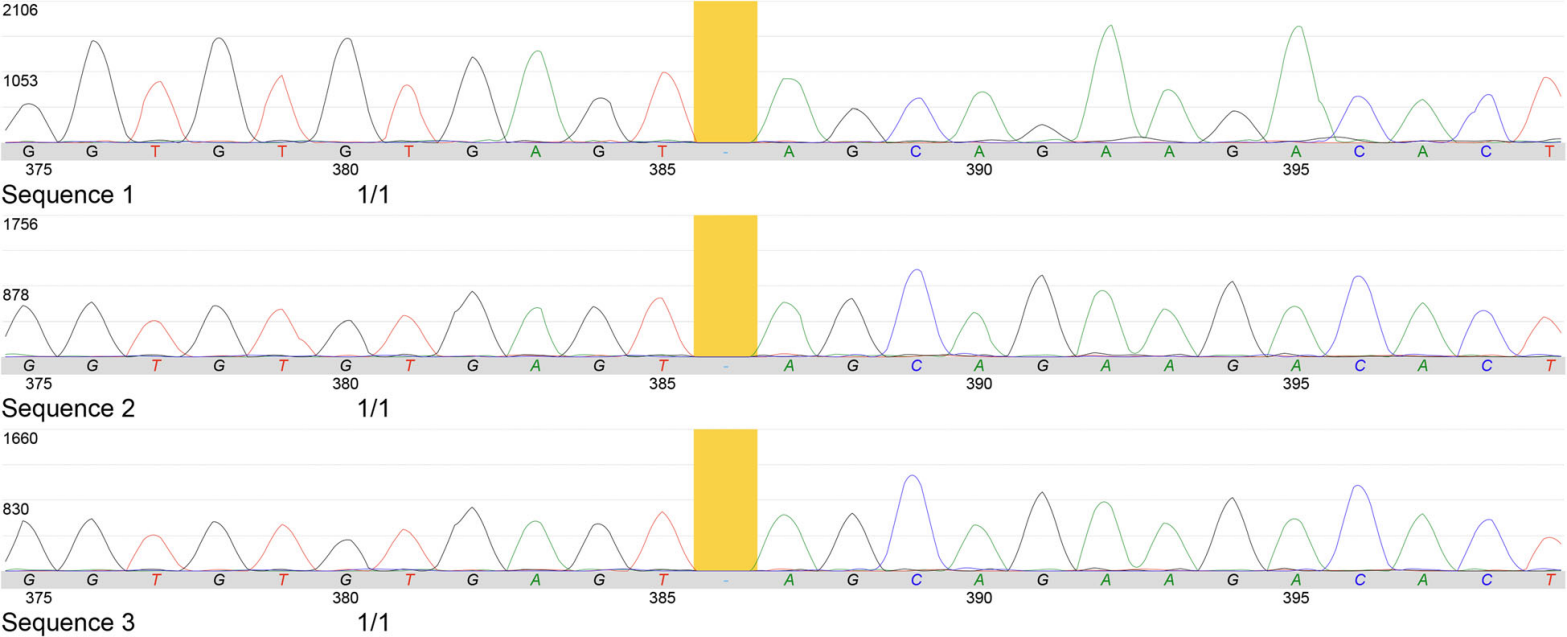
Graphical display of homozygous mutation in the *ZMYND10* gene. Homozygous mutation in the ZMYND10 gene for a sequence variant designated c.386delC, which is predicted to result in premature protein termination (p.Ser129)

The woman’s physical examination and hysterosalphingography revealed no pathology and her basal endocrine assessment on the second day of her menstrual cycle were as follows: FSH: 6.23 IU/L, LH: 3.37 IU/L, estradiol: 45.05 pg/mL, progesterone: 0.8 ng/mL, free triiodothyronine: 4.34 pmol/L. At the time she referred in our clinic she was 33 years old and a total of 14 antral follicles were detected in basal vaginal ultrasonography. Her husband, 36 years of age, had a history of total immotile spermatozoa with a mean sperm concentration of 7 million/mL (ranging between 2 and 9 million/mL), recurrent upper respiratory tract infections, dextrocardia (Fig. 1a) and situs inversus visceralis (Fig. 1b). Urogenital examination showed no pathology and endocrine parameters were as follows: FSH: 6.51 mIU/mL, LH: 8.71 mIU/mL, testosterone: 4.40 ng/mL. His previous semen analysis revealed severe oligoasthenoteratozoospermia according to World Health Organization (WHO) criteria [17] with total immotility. TEM analysis revealed complete absence of dynein arms between outer microtubule-doublet-subfibers A and B within the sperm tails in patient’s sample (Fig. 2a, arrowheads), whereas sperm tails presented normal dynein formation in internal control donor’s sample (Fig. 2b, arrows). Ultrastructural sperm tail organization other than dynein arms displayed normal morphology in terms of 9 + 2 microtubules and radial spokes. Sperm viability test indicated 54% viable spermatozoa in the ejaculate (Fig. 3). Test results indicated that the patient is homozygous in the *ZMYND10* gene (Fig. 4), heterozygous in the *ARMC4* and *DNAH5* gene mutations. Twenty-five oocytes were collected, and 22 mature (metaphase II, MII), two dysmorphic and 1 degenerated oocytes were obtained after enzymatic (Hyase × 10, Vitrolife, Göteborg, Sweden) and mechanical denudation (EZ-strip, Research Instruments, Cornwall, United Kingdom), 2 h after oocyte retrieval. Dysmorphic and degenerated oocytes were excluded from microinjection treatment. Semen parameters at the day of IVF showed oligozoospermia (5 million sperm/mL), total asthenozoospermia and teratozoospermia (0% normal morphology) according to Kruger’s strict criteria [26]. Ten normal fertilization patterns (2PN) were detected with the fertilization rate of 45.5% where all zygotes except 1 have proceeded to cleavage stage. Following the extended culture, 4 blastocycts were detected on day 5 and 6. One 4AB and one 2AA blastocytes [27] were transferred into the uterus with no complications during embryo transfer procedure. Endometrial double wall thickness was measured 12 mm with transabdominal ultrasonography. Luteal phase support was provided with daily vaginal progesterone. Remarkably, couple did not wish remaining two blastocysts to be cryopreserved, hence the embryos were discarded according to the related law. Ten days following the embryo transfer, β-human chorionic gonadotropin (β-hCG) was measured as positive and conforming ultrasound scan detected 3 amniotic cysts. Following a perinatal consultation, fetal reduction was not recommended. On routine fetal ultrasonographic evaluation, performed on 16th weeks and 4 days, dichorionic triamniotic triplet fetuses were present where fetus B and C were reported as monochorionic diamniotic twins with no significant pathological findings. Live birth of two girls and a boy following a Cesarean section was performed on 32nd weeks and 4 days in November 2016. Apgar scores (first/fifth minute), weight and height of the newborns were as follows: girl, 1700 g, 43 cm, Apgar 6/8, girl, 1940 g, 44 cm, Apgar 5/7, boy, 2140 g, 46 cm, Apgar 6/9. A short term pediatric intensive care hospitalization is needed because of preterm delivery; nevertheless couple with the newborns were discharged without any complication and abnormalities. Infants were 3 months old during the manuscript preparation with normal pediatric developmental rate.

## Discussion

Kartagener’s Syndrome is a disease in which ciliary ultrastructural morphology is deficient; consequently, patients suffer from chronic or recurrent upper respiratory diseases and infertility. The main feature that separates PCD from KS is the presence of situs inversus visceralis or dextrocardia in KS. Mouse studies have provided evidence that some genes play a critical role while determining the left-right differences in the body, by asymmetrical expression during development. They are crucial for the functions of ciliated cells within the embryonic organizer of gastrula stage embryos, therefore it is documented that rotation of nodal cilia (Hensen’s node) and the resulting uni-directional flow of extracellular fluid are required for establishing left–right differences [28, 29]. Axoneme is the main microtubular architecture in the flagella of the sperm and dynein arms function as the motor units as they are able to transform chemical energy into ATP to regulate microtubule sliding and mediate mechanical movement. Each dynein molecule forms a cross-bridge between two adjacent microtubules of the ciliary axoneme and the motor domain ATPases Associated with diverse cellular Activities (AAA) undergoes a conformational change that causes the microtubule-binding stalk and flagellar beating [30]. There are reports with the lack of radial spokes [31] or solely inner dynein arms [32–34] where infertility was evident because of asthenozoospermia, but motile respiratory cilia may be present. Clinical KS, with bronchiectasis, recurrent sinusitis and situs inversus were also reported to have motile sperm and respiratory cilium. In a couple with male KS, where spermatozoa were progressively motile, successful pregnancy has been reported using classical IVF, not using ICSI [5]. TEM investigation is suggested to be a gold standard on diagnosis of KS; therefore, specific mutations have already been described and considered to be more accurate for diagnosis [29]. Axonemal ultrastructure may appear normal in some cases [6, 8] which may not necessarily eliminate KS diagnosis. Reports indicating the management of infertility in KS cases are summarized chronologically in Table 1.

**Table 1:** A chronological summary of published cases on Kartagener’s syndrome and its management in the clinical context

Report	Prognosis	Management	Outcome
von Zumbusch et al. [4]	Case1: sperm concentration 75 × 10^6^/mL, Case2: sperm concentration 210 × 10^6^/mL, both total immotile	Diagnosed by eosin test and TEM, random sperm pick and fertilization by ICSI	Live birth of healthy twins (case 1) and a singleton (case2)
Abu-Musa et al. [35]	sperm concentration of 58 × 10^6^/mL, total immotile	random sperm pick in ICSI	No fertilization and pregnancy
Kay et al. [5]	Mean sperm concentration of 49 × 10^6^/mL, 25% mean motility	Diagnosed by TEM, fertilization by ICSI after gradient and swim-up	Live birth of a singleton male
Cayan et al. [14]	Case1: azoospermia after centrifugation, immotile testicular sperm Case2: sperm concentration of 4.8 × 10^6^/mL, < 5% viable in eosin test, total immotile	Case1: TESE, HOS test and ICSI Case2: TESE, eosin test (95% viability in testicular sperm), ICSI	Case 1: Birth of a singleton after frozen embryo transfer Case2: 4 embryos transferred with no pregnancy
Westlander et al. [15]	Case 1: severe oligozoospermia with total immotility Case2: normal sperm count and morphology with total immotility (63% viability with HOS test, absence of dynein arms by TEM.)	Case 1. 1st attempt: HOS test and ICSI Case1. 2nd attempt: TESE, HOS test and ICSI Case2: Fertilized by ICSI after HOS test with half sperm injected from ejaculate and other half from TESE	Case1. 1st attempt: No fertilization Case1. 2nd attempt: Live birth of twins Case2: Ongoing singleton pregnancy from one embryo transfer derived from testicular sperm
Aktan et al. [23]	Cases with total immotility	HOS test or tail laser shot before ICSI	Take home baby rate/cycle of 19% vs 5.9% when testicular sperm, and 28% vs 16.7% when ejaculated sperm by laser vs random selection, respectively
Kaushal et al. [16]	sperm concentration of 58 × 10^6^/mL, no motility, and 7% normal morphology	ICSI of partially motile sperm after TESE	Live birth of twins
Kordus et al. [37]	Total immotile sperm, 40% viability by eosin-nigrosin test, defects on dynein arms by TEM	ICSI following HOS test	Live birth of twins
Matsumoto et al. [7]	sperm concentration of 57.2 × 10^6^/mL, 30% viability by eosin test, 0.3% sperm motility	Absence of both dynein arms by TEM, fertilization by ICSI after swim-up	Live birth of a singleton baby
Nunez et al. [47]	sperm concentration of 1.2 × 10^6^/mL, 30% viability by eosin test, 0.3% sperm motility	HOS test to select ejaculated sperm for ICSI in 3 cycles, testicular sperm in 1 cycle using ICSI	Low grade or no cleavage embryo development, no pregnancy. Live birth after usage of donor sperm
Hattori et al. [11]	sperm concentration of 0.9 × 10^6^/mL, 54% viability by eosin test, no sperm motility	Absence of one or both dynein arms by TEM, pentoxifylline-activated sperm pick by ICSI	Live birth of a singleton baby
Vicdan et al. [48]	Azoospermia	Absence of dynein arms in nasal biopsy by TEM, fertilization by testicular sperm in ICSI	Live birth of a singleton baby
Geber et al. [36]	sperm concentration of 43 × 10^6^/mL, no sperm motility	HOS test to select viable ejaculated sperm during ICSI	Live birth of twins
McLachlan et al. [12]	sperm concentration of 10.1 × 10^6^/mL, 20% vitality, no sperm motility	Disorganized axoneme in TEM, Random testicular sperm retrieval during ICSI, assisted oocyte activation by calcium chloride rich medium	Live birth of a singleton baby
Ebner et al. [13]	sperm concentration of 1.8 × 10^6^/mL, 32% vitality, no sperm motility	Theophylline activation resulted in no motility, HOS test selected sperm used during ICSI, assisted oocyte activation by ionophore solution	Live birth of twins
Montjean et al. [10]	2 cases with immotile spermatozoa	Case1: non-progressive motility achieved after pentoxifylline activation in first cycle, HOS test used after no pentoxifylline activated sperm in second cycle, HOS test used in third cycle Case2: fertilization using sperm after HOS test in two cycles	Case1: No pregnancy in first two cycles, no fertilization in third cycle. Case2: Fertilization but no pregnancy in first cycle, live birth of a singleton after second cycle using vitrified oocytes remained from first cycle

Most of the cases with KS have been considered incurably infertile, until the availability of ICSI. With ICSI, not only patients with KS but also many cases with total asthenozoospermia were able to produce viable, but poorer quality embryos after random selection of immotile spermatozoa [3]. In our case, with the help of LAVA, which is introduced for the first time in literature for a diagnosed KS patient, it is shown that achievement of good quality embryos with a high implantation rate is possible if viable sperm can be selected. Von Zumbusch et al. firstly reported live birth in KS using ICSI, nevertheless total immotile sperms were picked randomly for injection and no methodological sperm selection was discussed in their report [4]. The disadvantage of randomly picked immotile spermatozoa for oocyte injection was discussed in the case report of Abu-Musa et al. where injection of four oocytes resulted in no fertilization [35]. The ultimate reason for low outcome may be that, motility is the most common method for selecting viable sperm during ICSI and in many cases of KS, motility is affected. Whatever the rate is, the motility in a sperm population is extremely important, as it is the unique marker of viability in sperm selection. Therefore, in these cases the probability of selecting a nonviable sperm for ICSI is relatively higher [14]. Many diagnostic viability tests are available, however only a few is used in wet preparations, during ICSI. The hypo-osmotic swelling test is the classical method to detect immotile but live spermatozoa in wet preparations [17], and live birth was reported with its usage in KS [36]. However, test can be considered detrimental, as the sperm cells are completely exposed to imbalanced osmotic conditions thus causing hypo-osmotic stress. Detection of sperm tail flexibility by mechanical touching using an ICSI pipette was also suggested to determine live spermatozoa. In this technique, sperm cells with a rigid tail which show a passive head displacement upon movement of the tail were considered to be non-viable [18, 19]. Alternatively, Aktan et al. have reported that, LAVA can also be successfully used to detect live but immotile spermatozoa in non-KS patients with testicular or ejaculated immotile spermatozoa. Besides, they demonstrated similar tail reaction patterns in HOS test and laser exposure, nevertheless reported significantly higher fertilization, cleavage and take-home-baby rates when laser is used [23]. Pipette touching technique needs to be validated for the axoneme abnormalities and the cellular mechanisms behind this phenomenon need to be clarified. It is not functionally related with LAVA application, as in LAVA, an instant dynamic movement is observed when laser is applied on the tail, probably due to a sudden irritation on membrane. Firstly with current report, we present a visual demonstration of the LAVA procedure and utilize it in a fully diagnosed KS patient.

It is logical to speculate that an axonemal pathology could alter fertilization, cell division and differentiation as centriole of the sperm plays a crucial role in mentioned processes. Extended culture of embryos to blastocyst stage, which were developed after LAVA has been reported before [37]. We believe in current case report, it is important to confirm that extended embryo culture in KS seems safe, meaning that sperm with axonemal abnormality has potential to maintain normal preimplantation embryonic development. In the light of this context, extended embryo culture is a valuable tool to reduce the number of embryos transferred, in order to avoid multiple pregnancies. In this case, the couple’s long history for achieving pregnancy and their refusal for cryopreservation led us decide for a double embryo transfer. Although assisted hatching was not performed before embryo transfer, which is speculated to increase monozygotic twining, it is obvious that one of the blastocysts had divided in the uterus and caused a triplet pregnancy. Risk factors that lead to monozygotic twinning after in vitro fertilization was identified in a recent report, where young oocyte age, extended culture, and year of IVF treatment cycle were found to be significantly associated [38]. When considered in the light of this data, except for the oocyte age, other two risk factors were present in this case, on the other hand guidelines clearly suggest that for patients with two or more previous failed fresh IVF cycles or with a less favorable prognosis (a severe male factor in this case), one additional embryo may be transferred according to individual circumstances [39]. With the satisfactory success rate of freeze-thaw cycles, we believe, especially for the blastocyst transfer, sequential single embryo transfer policy is the most favorable approach to reduce multiple pregnancies without lowering live birth rates [40]. According to current data and after a comprehensive PubMed search, we could not maintain any evidence to speculate that embryos with KS have increased risk for monozygote twinning, as hatching mechanism is not related to ciliary action.

Laser Assisted Viability Assessment has the potential to make infertility clinics avoid using testicular sperm from KS patients. As recent studies demonstrate, KS can be resulted from many different mutations which are obviously not limited to the ejaculated spermatozoa, but the same mutations affect sperm development and maturation in seminiferous tubules as well. For this reason we believe that, testicular spermatozoa in KS would hardly be advantageous from ejaculated spermatozoa, yet more comparative studies are needed. There are reported live births after testicular sperm injection in KS males [14, 16], nonetheless we believe that KS is not an indication for testicular sperm extraction unless azoospermia or a post-testicular pathology exist.

Previous reports indicate gene mutations or deletions such as *DNAI1, DNAI2, DNAH5, DNAH11, CCDC103, ARMC4, KTU/DNAAF2, LRRC50/DNAAF1, LRRC6, DYX1C1, ZMYND10, CCDC39, CCDC40, CCDC164, HYDIN, RSPH4A* and *RSPH1* in published cases of PCD and KS [41]. Having the history of recurrent respiratory tract infections and dextrocardia in this patient, we performed a genetic mutation screening and TEM analysis to confirm the KS diagnosis. TEM evaluation demonstrated an apparent global loss in both dynein arms and NGS revealed a homozygous mutation in the *ZMYND10* gene. It has been reported that individuals with biallelic truncating variants in *ZMYND10* were found to have primary ciliary dyskinesia with or without laterality defects, and lacking both inner and outer dynein arms observed by TEM evaluation [42, 43].

This patient was heterozygous in the *ARMC4* gene, which is predicted to result in the amino acid substitution p.Gly781Val. This variant is listed in public databases with an allele frequency as high as 0.35%, which is likely too common to be the primary cause of the disease. The amino acid residue p.Gly781 of the *ARMC4* protein has been conserved during evolution. Homozygous or compound heterozygous pathogenic variants in *ARMC4* are reported in individuals with reduced number of outer arms and ciliary beat frequency [44]. A second plausible pathogenic variant in *ARMC4* was not detected which can explain autosomal recessive primary ciliary dyskinesia. On the other hand, laboratory was not able to sequence four coding exons (exon 2, 8–10) in *ARMC4* due to a very high level of sequence identity elsewhere in the genome. To our knowledge no documented pathogenic variants have been reported in these 4 exons [45]. Although we suspect that this variant is too common to be the primary cause of the disease, without additional information we classify it as a variant of uncertain significance.

This patient was also heterozygous in the *DNAH5* gene for a rare missense variant defined as c.1715 T > G (Leu572Trp). This variant is listed in public database with an allele frequency of ~ 0.1%. DNAH5 is a large protein with over 4600 amino acids. Undocumented and rare (allele frequency < 0.01) missense variants in *DNAH5* are commonly found in presumably healthy individuals, making interpretation of rare missense variants difficult. Biallelic pathogenic variants in *DNAH5* are documented to cause autosomal recessive primary ciliary dyskinesia [46].

To conclude, this case report firstly presents a successful diagnosis and non-invasive management of male Kartagener’s Syndrome, resulted in birth of healthy triplets presented with a monozygotic twinning. Laser assisted viability assessment allows a practical and effective selection of viable spermatozoa during ICSI set up in cases of total asthenozoospermia. Embryo development and implantation are not negatively affected neither with the usage of LAVA nor of the sperm with impaired axoneme.

## Abbreviations

HOST: Hypo osmotic swelling test
ICSI: Intracytoplasmic sperm injection
IVF: In vitro fertilization
KS: Kartagener’s syndrome
LAVA: Laser assisted viability assay
MOPS: 3-(N-morpholino) propanesulfonic acid
NGS: Next generation sequencing
PCD: Primary ciliary dyskinesia
TEM: Transmission electron microscopy
WHO: World Health Organization
